# Short-Term Behavior of Slag Concretes Exposed to a Real In Situ Mediterranean Climate Environment

**DOI:** 10.3390/ma10080915

**Published:** 2017-08-08

**Authors:** José Marcos Ortega, Isidro Sánchez, Marta Cabeza, Miguel Ángel Climent

**Affiliations:** 1 Departamento de Ingeniería Civil, Universidad de Alicante, Ap. Correos 99, 03080 Alacant/Alicante, Spain; isidro.sanchez@ua.es (I.S.); ma.climent@ua.es (M.Á.C.); 2 Grupo ENCOMAT, Escola de Enxeñaría Industrial, Universidade de Vigo, Campus Universitario, 36310 Vigo, Spain; mcabeza@uvigo.es

**Keywords:** ground granulated blast-furnace slag, sustainability, real condition exposure, Mediterranean climate environment, curing, temperature, relative humidity, ordinary Portland cement

## Abstract

At present, one of the most suitable ways to get a more sustainable cement industry is to reduce the CO_2_ emissions generated during cement production. In order to reach that goal, the use of ground granulated blast-furnace slag as clinker replacement is becoming increasingly popular. Although the effects of this addition in the properties of cementitious materials are influenced by their hardening conditions, there are not too many experimental studies in which slag concretes have been exposed to real in situ environments. Then, the main objective of this research is to study the short-term effects of exposure to real Mediterranean climate environment of an urban site, where the action of airborne chlorides from sea water and the presence of CO_2_ are combined, in the microstructure and service properties of a commercial slag cement concrete, compared to ordinary Portland cement (OPC). The microstructure was studied with mercury intrusion porosimetry. The effective porosity, capillary suction coefficient, chloride migration coefficient, carbonation front depth, and compressive strength were also analyzed. Considering the results obtained, slag concretes exposed to a real in situ Mediterranean climate environment show good service properties in the short-term (180 days), in comparison with OPC.

## 1. Introduction

Today, the cement industry still needs to be more environmentally sustainable and one of the most suitable ways to reach this goal is to reduce the CO_2_ emissions generated during cement production. In relation to that, the use of supplementary cementitious materials or additions as clinker replacement is becoming more and more popular [1,2,3,4,5,6,7,8,9,10,11]. This also has an added value from the environmental point of view, because their use entails lower energy consumption during the cement manufacture and, in addition to this, many of those supplementary cementitious materials are wastes from other industries whose reuse would partially solve the problem of their disposal.

Among those additions, the ground granulated blast-furnace slag (GGBS) and its influence on the microstructure and properties of cement-based materials has been well-studied, especially when the hardening of those materials has been produced in optimum laboratory conditions [12,13,14], in which they showed better service properties than ordinary Portland cement (OPC) [12]. This result is a consequence of the slag hydration reactions, which produce new CSH phases, making a more refined microstructure [4,12,15]. This pore refinement improves the properties of cement-based materials, such as their permeability [16] and chloride ingress resistance [17,18,19].

On the other hand, there are several studies in which slag cement-based materials were exposed to non-optimum laboratory hardening conditions, with constant combined temperature and relative humidity [4,14,20,21,22]. They are a good approach to real hardening environmental conditions, and the behavior of slag mortars and concretes was adequate in general [4,21,22], especially in environments with relatively high temperature and relative humidity [14,21].

Nevertheless, there are not many experimental studies in which slag concretes have been exposed to real in situ environments. In that regard, the work made by Thomas et al. [18] should be noted, in which concrete containing ground pelletized blast-furnace slag was evaluated after 25 years of exposure in a marine tidal zone, placed on the Thames Estuary near Shoeburyness in Essex (UK). As findings of that research [18], it is important to note that the slag concretes showed significantly greater resistance to chloride ion penetration and it was concluded that the use of slag at relatively high levels of replacement resulted in a significant increase in the performance of concrete in a very aggressive marine environment.

In line to the abovementioned work [18], it is interesting to emphasize the research carried out by Scott et al. [23] in which a series of concrete blocks containing fly ash and slag were studied. These blocks were retrieved in 2004 from the US Army Corp of Engineers test site at Treat Island, Maine (USA) after 25 years exposure to a marine tidal environment, and they were tested to establish the depth of chloride penetration and to determine the chloride permeability and chloride diffusion coefficient. The results of this work [23] pointed out significant improvements in chloride resistance in the concretes with slag and fly ash, in comparison with control mixes without these additions. Nevertheless, a reduction of the resistance of the surface to scaling by the presence of fly ash and slag has been noted.

With respect to the works made by the Slag Research Institute of Duisburg-Rheinhausen (Germany), it is worth mentioning one of them [17], which consisted of studying the influence of slag content in the capillary porosity of concrete exposed to a real in situ environment. In that work [17], concrete blocks prepared using different binders of ordinary Portland cement and GGBS and water to cement ratios, were exposed to real outdoor environmental conditions, but protected from the rain. Before that, the blocks were cured during 7 days at 20 °C and 100% relative humidity in a chamber. After 12 years of exposure, the binders with higher contents of slag showed very low capillary porosities [17] compared to control binders.

Another study related to the topic of the present manuscript is that performed by Shattaf et al. [24], which investigated the effect on pore structure of concretes made with combinations of Portland cement, slag, and silica fume when exposed to hot and dry environments. This work [24] included the exposure of the materials to a real outdoor environment in Dubai, representing a typical hot climate. These authors [24] observed that hot climates seemed to have adverse effects on the pore structure characteristics of studied concretes and as a consequence, the properties of slag concrete mixes exposed to hot/dry environments, especially those without any water curing, were very poor.

In a recent study carried out by Pasupathy et al. [25], the carbonation resistance of two geopolymer concretes was investigated after being exposed to outdoor field conditions during eight years in Campbellfield, Victoria (Australia). These geopolymers were prepared using different binders of GGBS and fly ash, and their experimental results were compared to those obtained for conventional OPC and fly ash concretes exposed to similar conditions. In addition to the carbonation resistance, water absorption, sorptivity, total porosity, and differential pore size distribution were analyzed. As a conclusion of this research [25], the authors indicated that the carbonation rate of geopolymer concrete highly depends on the mix binder design of materials, although concrete with higher slag content showed similar resistance against carbonation compared to OPC concrete.

Finally, in relation to real structures made with slag cements, it is interesting to mention the 12 km of bridges of King Fahd Causeway (Saudi Arabia), which were built with Portland blast furnace slag cement, and nine years after completion, they were in excellent condition without any sign of reinforcement corrosion [12]. Other examples are the marine structures built along the North Sea coast in The Netherlands [26], most of them made using GGBS cement. In accordance to the field investigations of Polder et al. [26], in which several of those structures with ages between 18 and 41 years were studied, the majority of them showed no corrosion damage.

According to the results of the previously referenced experiences of exposure of slag cement-based materials to real in situ environments, it can be concluded that they showed different behavior depending on the climate conditions of the location where the samples were placed. Furthermore, there are not experimental studies about the behavior of GGBS cement-based materials hardened in the real conditions of the Mediterranean climate.

Therefore, the main objective of this research is to study the short-term effects of exposure to the real Mediterranean climate environment of an urban site, where the action of airborne chlorides from sea water and the presence of CO_2_ are combined, in the microstructure, durability and mechanical properties of concretes prepared with a commercial GGBS cement. Their behavior was compared to that observed for ordinary Portland cement (OPC) concretes. The study of the mixed effects of airborne chlorides and CO_2_ in slag concretes could be useful to design road infrastructures with high levels of traffic near the sea coast, as well as maritime structures or zones of the ports accessible to transit vehicles, as logistics areas, exposed to Mediterranean climate conditions. Moreover, for both slag and OPC concretes, the influence of curing was also studied, which could also be interesting in relation to in situ cast and precast concrete elements. Several abovementioned studies [24], in which slag concretes have been exposed to real environments, analyzed the effect of curing too, and it was concluded that an adequate curing was beneficial for the future performance of slag concrete elements. Regarding the experimental techniques used in the present work, the microstructure has been studied with mercury intrusion porosimetry. On the other hand, the analyzed durability-related parameters were the effective porosity, capillary suction coefficient, non-steady state chloride migration coefficient, and carbonation front depth. Finally, in order to check the mechanical performance of the concretes, the compressive strength was also determined.

## 2. Materials and Methods

### 2.1. Sample Preparation

The tests were performed in concrete samples, prepared using two different cement types. The first one was an ordinary Portland cement, designated CEM I 42.5 R (CEM I hereafter) according to the European standard UNE-EN 197-1 [27]. The second one was a cement which contributes to sustainability, a type III/B 42.5 L/SR [27] (CEM III from now on), with a content of GGBS between 66% and 80% of total binder. It has to be emphasized here that both cements used were commercial cements, in order to reproduce the real conditions of in situ construction and—especially—the difficulty of mixing cement and additions in the field. The different components of each one of commercial cements and its percentage of the total binder are detailed in Table 1. On the other hand, concrete samples were prepared using the dosage shown in Table 2.

Two kinds of cylindrical specimens were prepared for the different test methods. On one hand, cylinders were cast in moulds of 10 cm diameter and 15 cm height. On the other hand, samples with 15 cm diameter and 30 cm height were also made. After setting, both types of samples were maintained in 95% RH chamber and 20 °C for 24 h. Before being exposed to the different hardening conditions, the 15 cm height cylindrical samples were cut into cylinders of 5 cm thick. Finally, the tests were performed at 28, 90, and 180 days of age.

### 2.2. Environmental Conditions

The samples were divided in three groups depending on their curing and hardening conditions. The first group of samples was exposed to an optimum laboratory condition, of 20 °C and 100% relative humidity (RH). This condition was taken as a reference in order to compare the influence of real Mediterranean conditions in the microstructure and properties of the studied concretes.

**Figure 1:**
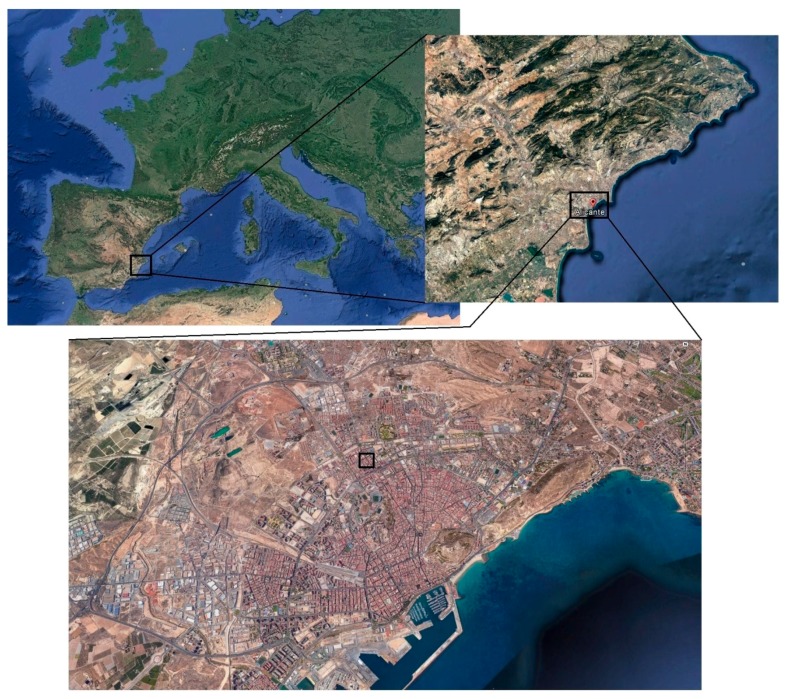
Location of the real exposure site (black line squares). The samples were placed on the flat roof of a building in the city center of Alicante, which is situated in the southeast of Spain. The distance between this exposure site and the Mediterranean Sea coastline was about 2.6 km. The satellite images were obtained using the Google Earth software (Google, CA, USA).

The second and the third group of samples were placed on the flat roof of a building in the city center of Alicante (Spain), close to one of the main streets of the city in an area of dense traffic (see Figure 1), so a high amount of CO_2_ was present in the environment. Furthermore, the exposure site was located at 2.6 km approximately from the Mediterranean Sea coastline, so the airborne chlorides coming from sea water could also have an influence in the behavior of the studied materials. In view of that, the location of the samples combined the characteristics of exposure classes IIb and IIIa defined by the Spanish Code on Structural Concrete EHE-08 [28], which would be equivalent to exposure classes XC3 (corrosion induced by carbonation with moderate humidity) and XS1 (corrosion induced by chlorides from sea water without direct contact with it) established in the Eurocode 2 [29].

**Figure 2:**
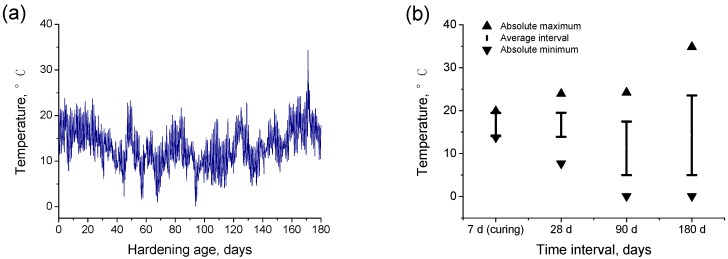
(**a**) Temperatures registered during the exposure period; (**b**) Absolute maximum, absolute minimum and interval of average temperatures at which the samples were exposed during the seven-day curing period and for those samples tested at 28, 90, and 180 days, respectively.

**Figure 3:**
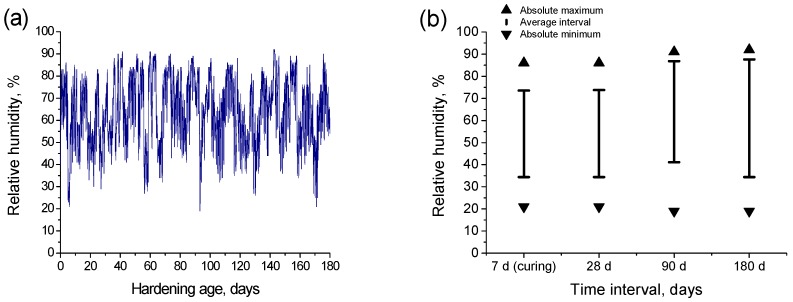
(**a**) Relative humidity registered during the exposure period; (**b**) Absolute maximum, absolute minimum and interval of average relatives humidity at which the samples were exposed during the seven-day curing period and for those samples tested at 28, 90, and 180 days respectively.

The highest exposure time period was 180 days and it covered the months from October to April. During this period, the environmental temperature and relative humidity in the exposure site were measured each 30 min. The temperatures registered during the exposure period can be observed in Figure 2a. The absolute maximum, absolute minimum, and interval of average temperatures at which the samples were exposed depending on the testing age are represented in Figure 2b. As can be observed, the temperatures showed a high variability, especially for the samples tested at 180 hardening days, which were exposed to temperatures ranged from 0 °C to 35 °C, although the average temperature for that time period was about 15 °C. On the other hand, the relative humidity measured along the exposure period is depicted in Figure 3a, and the absolute maximum and minimum of this parameter, as well as the interval of its average values registered at each testing period, are represented in Figure 3b. The relative humidity also showed a wide variation interval, which covered values between 20% and close to 90%.

Finally, in order to study the influence of curing for the specimens hardened in a real in situ Mediterranean environment, the second group of samples were watered twice a day during the first seven exposure days, while no additional curing was provided for the third group of samples. 

### 2.3. Mercury Intrusion Porosimetry

The microstructure of the concretes was studied using the classical mercury intrusion porosimetry, despite its drawbacks [6,7,30,31]. The tests were performed with a porosimeter model Autopore IV 9500 from Micromeritics (Norcross, GA, USA). Before the test, samples were oven dried for 48 h at 50 °C. For each testing age, two measurements were performed on each material. Total porosity, pore size distribution, and percentage of Hg retained at the end of the experiment were studied. The testing ages were 28, 90, and 180 days.

### 2.4. Capillary Absorption Test

The capillary absorption test was performed according to the standard UNE 83982 [32], which is based on the Fagerlund method to determine the capillarity of concrete. Cylinders of 10 cm diameter and 5 cm thickness were tested.

Regarding the selected pre-conditioning procedure, in the first place the specimens were completely dried in an oven at 105 °C for 12 h. Afterwards, until the beginning of the test, they were kept in a hermetically sealed container with silica gel during the next 12 h [14,33]. The reason for using this pre-conditioning procedure instead of imposing a water saturation degree of 70% on the specimens, as suggests the Rilem recommendation TC 116-PCD [34], was the long time that this would imply. This could cover up the effects of the hardening environment and age, because the contact of the concrete samples with water for a long time period would modify the hydration degree of the cement-based materials, affecting the consequent development of their durability-related properties.

Before the test, the circumferential surface was sealed using self-adhesive tape [34]. After that, the samples were introduced in a container with a flat base, according to the UNE 83982 [32]. The container was filled with distilled water until 5 ± 1 mm on the lateral surface and more than a 95% of the base of the sample was in contact with water. During the test, water level was kept constant and the container was hermetically closed. Samples were weighed at different times set in the standard. The test finished when the difference between two consecutive weights, with 24 h difference, was lower than 0.1% in mass. The effective porosity and the capillary suction coefficient were calculated according to the expressions

(1) $$\varepsilon_{e}\;=\;\frac{Q_{n}{\;-\;Q}_{0}}{A\cdot h\cdot \delta_{a}}$$

(2) $$K\;=\;\frac{\delta_{a}\cdot \varepsilon_{e}}{10\cdot \sqrt{m}}\;\mathrm{with}\;m\;=\;\frac{t_{n}}{h^{2}}$$

where ε_e_ is the effective porosity, Q_n_ is the weight of the sample at the end of the test (g), Q_0_ is the weight of the sample before starting the test (g), A is the surface of the sample in contact with water (cm^2^), h is the thickness of the sample (cm), δ_a_ is the density of water (1 g/cm^3^), K is the capillary suction coefficient (kg/m^2^min^0.5^), m is the resistance to water penetration by capillary suction (min/cm^2^), and t_n_ is the time necessary to reach the saturation (min).

For each cement type and condition, three different samples were tested at each age.

### 2.5. Forced Migration Test

The forced chloride migration test was performed on water-saturated mortar samples, according to NT Build 492 [35] whose main result is the non-steady-state chloride migration coefficient D_NTB_. For each cement type and hardening condition, three different cylindrical specimens of 10 cm diameter and 5 cm height were tested.

### 2.6. Carbonation Front Depth

The carbonation front depth was measured in cylindrical samples of 15 cm diameter and 30 cm height, once they were used for determining the compressive strength. Superficial pieces of these samples were sprayed with phenolphthalein and the carbonation front depth was measured from the limit between the colorless and pink-colored areas. For each cement type and hardening condition, the carbonation front depth was gathered in several pieces taken from three different cylindrical samples of 15 cm diameter and 30 cm height.

### 2.7. Compressive Strength

The compressive strength was measured according to the Spanish standard UNE EN 12390-3 [36]. Three different 15 cm diameter and 30 cm height cylindrical specimens were tested for each cement type and hardening condition.

## 3. Results

### 3.1. Mercury Intrusion Porosimetry

**Figure 4:**
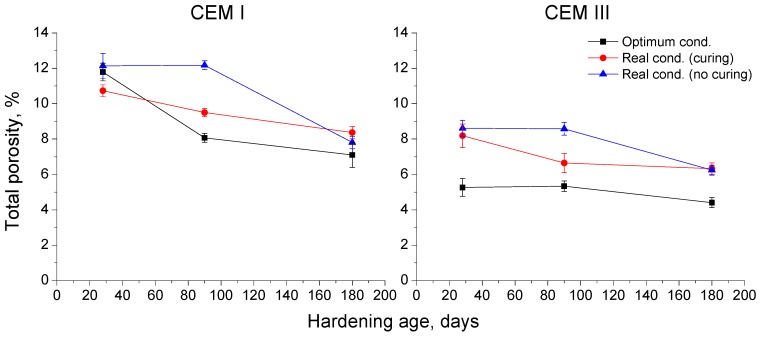
Results of total porosity for CEM I and III concrete specimens.

The results of total porosity for CEM I and III concretes are depicted in Figure 4. At 28 days, this parameter was very similar for all CEM I specimens. From then until 180 days, the total porosity decreased for CEM I concretes hardened in the optimum condition, mainly from 28 to 90 days (black line in Figure 4), and for those exposed to the real condition and cured during the first 7 exposure days (red line in Figure 4). However, for CEM I samples kept in the real environment without the initial seven days of curing (blue line in Figure 4), this parameter remained practically constant between 28 and 90 days, and fell until 180 days. At that age, similar total porosities were observed for CEM I concretes, independently of their hardening condition.

On the other hand, the lowest total porosities for CEM III concretes were noted in the optimum condition, which kept practically constant during the study period. At 28 and 180 days, CEM III samples hardened in the real environment showed the same total porosity values regardless of whether or not they were cured. Nevertheless, for those CEM III specimens, the evolution of this parameter was influenced by the initial curing, because for those cured during the initial seven exposure days, the total porosity mainly decreased between 28 and 90 days, and for those without this curing, this parameter fell mostly from 90 to 180 days. Comparing both types of cement, it is important to note that the lowest total porosities corresponded to CEM III concretes at all ages for each hardening condition.

**Figure 5:**
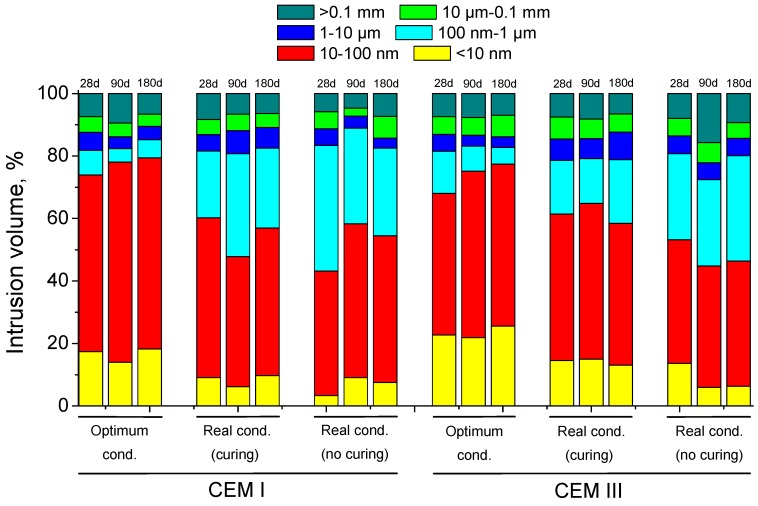
Pore size distributions for CEM I and III concretes obtained for each hardening condition.

The pore size distributions for CEM I and III specimens are shown in Figure 5. The most refined microstructure for CEM I concretes was observed in optimum condition, and it became even more refined with time. Regarding CEM I samples exposed to a real environment, at 28 days the microstructure refinement was higher for those cured during the initial seven exposure days. Despite that, the porous network of these samples hardly changed with time and at 180 days they were very similar to observed at 28 days. For non-cured CEM I specimens, in spite of their less refined microstructure at 28 days, the percentage of finer pores increased until 90 days, showing scarce difference at 180 days compared to pore size distribution of CEM I cured specimens.

For CEM III concretes, as happened for CEM I ones, those hardened in optimum condition showed the most refined porous network compared to the other conditions. In relation to CEM III specimens kept in real environmental condition, the microstructure was less refined for those non-cured at all ages. Moreover, it was observed a loss of porous network refinement with time for these CEM III samples exposed to real condition, for cured samples this loss has mainly been noted between 90 and 180 days, and for non-cured samples it was observed earlier, from 28 and 90 days. In general, for each condition the microstructure refinement was higher for CEM III concretes than for CEM I ones, as indicated by the higher percentage of smaller pore fraction (<10 nm), except for non-cured specimens exposed to real condition, whose porous network at 90 and 180 days was less refined for CEM III than for CEM I.

**Figure 6:**
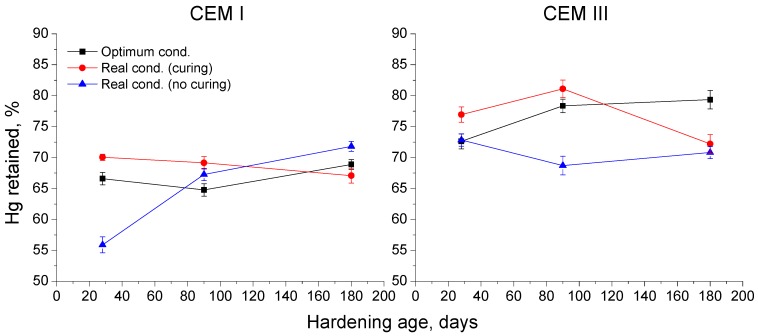
Results of mercury retained at the end of MIP tests for CEM I and III concrete samples.

The results of percentages of Hg retained in the samples at the end of the experiment are depicted in Figure 6. For CEM I, concretes exposed to optimum condition this parameter kept practically constant during the study period, as also happened with those hardened in the real condition and cured during the first seven exposure days. On the other hand, for non-cured CEM I specimens kept in the real condition, the Hg retained was relatively low at 28 days, although it started to rise after that, reaching a similar value at 180 days to those observed for the other conditions.

The Hg retained increased with time for CEM III concretes exposed to optimum condition. Different tendencies of this parameter were observed for CEM III specimens hardened in the real conditions. For cured samples, Hg retained rose between 28 and 90 days, and fell afterwards. For non-cured ones, this parameter kept practically constant. At 180 days, Hg retained was very similar for CEM III samples exposed to the real condition, independently of curing. In general, in the long-term CEM III concretes showed higher values of Hg retained than CEM I ones.

### 3.2. Capillary Absorption Test

**Figure 7:**
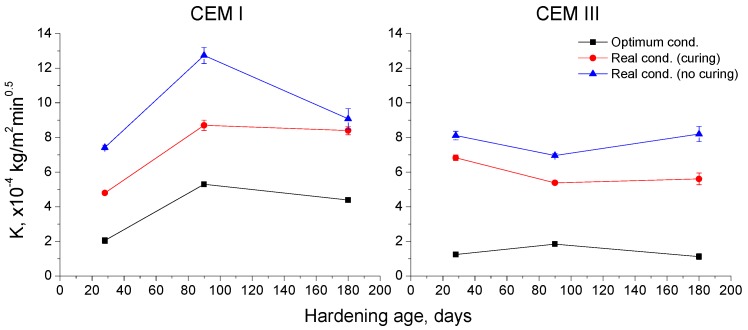
Results of capillary suction coefficient (K) for CEM I and CEM III concrete samples.

The parameters obtained from this test, as mentioned previously, are the capillary suction coefficient K and the effective porosity of the samples. The results of the capillary suction coefficient K can be observed in Figure 7. This parameter increased from 28 and 90 days for CEM I samples and decreased or kept practically constant from then until 180 days. At that time, the highest coefficient K values were noted for CEM I specimens exposed to real conditions. This coefficient hardly changed with age for CEM III samples, and the lowest values were observed for those specimens kept in optimum condition and the highest corresponded to those non-cured hardened in the real condition. Moreover, at 180 days, the coefficient K was lower for CEM III samples than for CEM I ones.

**Figure 8:**
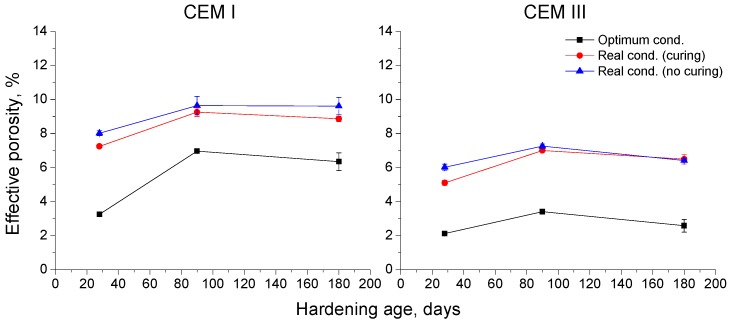
Results of effective porosity for concretes made with CEM I and CEM III.

The effective porosity results are depicted in Figure 8. Again for each environment, the lowest values of this parameter were observed for CEM III samples. The effective porosity showed a slight increase for all samples between 28 and 90 days, and kept constant or hardly decreased from 90 and 180 days. Furthermore, the effective porosity for each cement type was very similar for samples exposed to the real conditions, regardless of curing, and higher than that observed for those maintained in optimum condition.

### 3.3. Forced Migration Test

**Figure 9:**
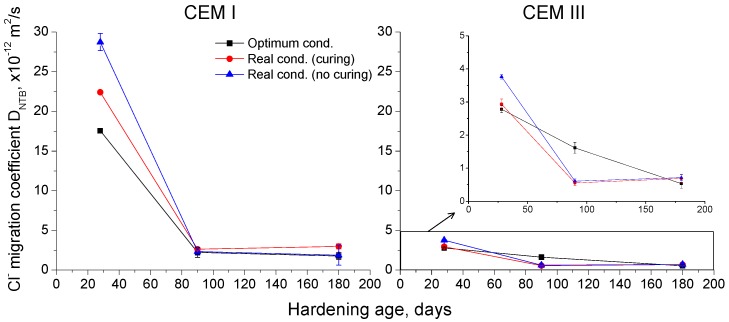
Results of non-steady-state chloride migration coefficient for concretes prepared using CEM I and CEM III, respectively.

The results of the non-steady-state chloride migration coefficient D_NTB_ obtained for CEM I and III concretes are depicted in Figure 9. The lowest migration coefficients were observed for CEM III samples, especially in the short-term. The reduction of this parameter was mainly produced between 28 and 90 days, particularly for CEM I specimens. In the long-term, the difference between both types of concretes was reduced, but the migration coefficient was still lower for CEM III samples than for CEM I ones. For both cement types, this coefficient was lower for samples exposed to optimum conditions, although at 180 days it was very similar for all conditions.

### 3.4. Carbonation Front Depth

**Figure 10:**
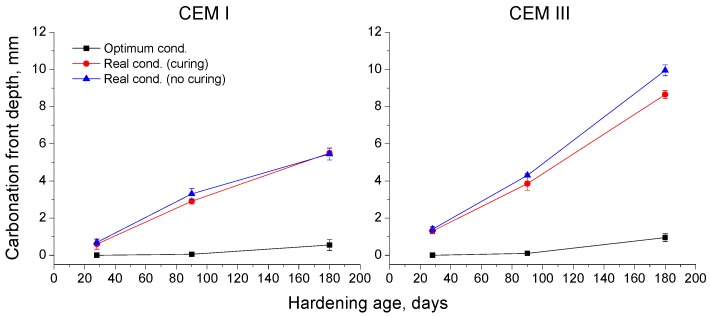
Evolution of carbonation front depth for CEM I and III specimens.

The changes with time of carbonation front depth for studied concretes can be observed in Figure 10. For both types of cement, the lowest carbonation front depths were noted for samples exposed to optimum conditions, and they kept practically constant during the study period. On the other hand, for samples hardened under the real conditions, the carbonation front depths increased with time, independently of curing, and at 180 days these carbonation depths were higher for CEM III samples than for CEM I ones.

### 3.5. Compressive Strength

**Figure 11:**
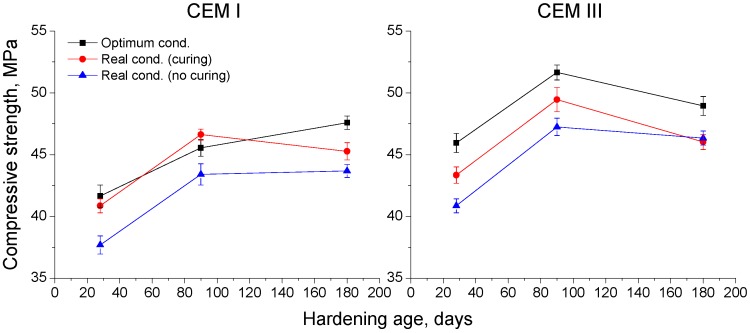
Results of compressive strength for concretes prepared using CEM I and CEM III respectively.

The results of compressive strength obtained for CEM I and III concretes are depicted in Figure 11. For CEM I samples hardened in the optimum conditions, the compressive strength increased between 28 and 180 days, and for those exposed to a real environment this parameter rose only from 28 to 90 days, and kept practically constant since then until 180 days. For this cement type, in general the highest compressive strengths were observed for the optimum condition and the lowest corresponded to non-cured samples hardened in the real condition.

For CEM III samples, the compressive strength increased from 28 to 90 days, and decreased since then until 180 days for all conditions. At 28 and 90 days, the highest values of this parameter were noted for the optimum condition, and the lowest compressive strengths were observed for non-cured samples exposed to the real environment. Nevertheless, at 180 days, the samples hardened in the real condition showed practically the same strength, regardless of curing, while those kept in the optimum environment continued showing the highest value of this parameter.

Regarding the comparison between CEM I and III concretes, at 28 and 90 days, the compressive strength was higher for CEM III samples under all conditions, although the values of this parameter at 180 days were similar for both cement types or slightly higher for CEM III specimens.

## 4. Discussion

### 4.1. Microstructure Characterization

Regarding the mercury intrusion porosimetry results, for each condition the concretes made with CEM III showed lower total porosities (see Figure 4) compared to those prepared using CEM I along the studied time period. This could be due to the effects of slag hydration, which produced additional solid phases, reducing the volume of pores of the sample [4,12]. For CEM I samples, the total porosity at 28 days was very similar independently of environmental and curing conditions. This fact could be related to the relatively high average temperatures registered throughout the 7-day additional curing period and the first 28 exposure days (see Figure 2b), which ranged from 13 to 20 °C approximately, so they did not differ too much in comparison with the 20 °C temperature of the optimum laboratory condition. On the other hand, for CEM III samples the difference at 28 days among total porosity values of those samples kept in the optimum conditions and those hardened in the real environment was more noticeable. This different behavior between CEM I and III could be explained in relation to the higher sensitivity of slag hydration to hardening temperature [4,21]. As has been noted, the temperature was slightly lower for the real environment over the initial 28-day period, so this could have slowed down the development of slag hydration, and the consequent formation of new solid phases, entailing a greater porosities in the very short-term.

In general, at 90 and 180 days, the lowest total porosity values were observed for the samples kept under the optimum laboratory condition, independently of the cement type used, although this was more significant for CEM III samples. This optimum condition provided high amount of water, which would facilitate the development of clinker and slag hydration, producing a quicker formation of solid phases [4,22] and porosity reduction.

The total porosity evolution of CEM I and III concretes hardened under the real in situ environment showed differences depending on the curing process. First of all, a progressive decrease of this parameter was noted from 28 to 180 days for those samples which were cured along the first 7 exposure days, while for those where 7-days additional curing period was not provided, the total porosity kept constant between 28 and 90 days, and decreased since then up to 180 days. This delay in the total porosity reduction for non-cured specimens could be related to the lower availability of water, which would imply a slower development of clinker and slag hydration [4]. Then, these specimens would need more time to reach similar values of this parameter compared to cured ones, as would suggest that practically no differences of total porosity between cured and non-cured samples were observed at 180 days for both analyzed cement kinds.

In relation to pore size distributions (see Figure 5), the most refined microstructure corresponded to the optimum laboratory condition for both studied cement types. This result is in accordance with total porosity ones, and would reveal the beneficial effects of a condition with very high humidity combined with high enough temperature in the development of slag and clinker hydration [4,21,22]. The real conditions, with and without seven days of additional curing, brought a less refined pore network of the studied samples, probably due to the lower temperature and relative humidity, which would make the development of hydration reactions of clinker and slag more difficult and slower [4]. For CEM I concretes exposed to the real conditions, the initial 7-day curing had noticeable effects at 28 days, but after 180 days their pore size distribution hardly showed differences, notwithstanding the curing procedure. On the contrary, the effects of this initial seven-day curing were more significant for CEM III samples, producing a more refined microstructure compared to non-cured ones throughout the studied period. As a consequence, it seems that the lower availability of water, in combination with lower environmental temperatures, would affect more on slag concretes. In addition to this, it was noted a loss of pore refinement with time for those slag cement samples exposed to real environment, which could be caused by the possible formation of shrinkage cracking, due to the lower humidity of this condition [4,37], although no effect was observed in total porosity as has been previously explained. However, overall the CEM III samples showed higher pore refinement than the CEM I ones, with the exception of those exposed to the real environment without additional curing.

With respect to the Hg retained in the sample once the mercury intrusion porosimetry test has finished (see Figure 6), it provides information about the tortuosity of the pore structure [38]. This parameter showed slight variations with time for CEM I samples hardened in the optimum condition which is in keeping with their pore size distributions. Moreover, for this cement type, the lowest values of Hg retained were obtained at 28 days for samples exposed to the real environment without 7 days of additional curing, which could mean a low tortuosity of the pore network, showing the effects of non-optimum environmental temperature and relative humidity in clinker hydration [4]. The tendencies of Hg retained for CEM III samples generally coincided with the evolution of their pore size distributions. The slag cement concretes kept in the optimum condition showed a continuous rise of this parameter, which would indicate an increase of the pore network tortuosity, that is, a progressive microstructure refinement, which would agree with the other mercury intrusion porosimetry results already discussed. For CEM III specimens hardened in the real condition, a fall of the Hg retained was noted from 28 to 180 days, suggesting a loss of pore structure tortuosity. This decrease was mainly produced between 90 and 180 days for cured CEM III samples and since 28 until 90 days for non-cured ones, which would also coincide with the loss of pore refinement for these samples revealed by their pore size distributions previously explained. Nevertheless, according to results of Hg retained, in general the tortuosity of the pore network for CEM III concretes was higher than for CEM I ones, which again would be in agreement with the results of pore size distributions.

### 4.2. Durability-Related Parameters

First of all, the capillary suction coefficient K is related to the ingress of water in concrete structures and, as a consequence, to the ingress of aggressive substances, which can produce the corrosion of steel in reinforced and prestressed structures. For both analyzed cement types, the lowest values of coefficient K were observed for the optimum condition (see Figure 7), and the highest ones for the real condition in which no additional seven-day curing was supplied, which overall would be in keeping with microstructure characterization results. The optimum condition would favor the development of clinker and slag hydration, due to its elevated relative humidity and high enough temperature [4,21,22], producing that the capillary suction coefficient K was lower compared to the real environment. For that in situ environment, the lower humidity available and the smaller average temperature values would slow down the abovementioned hydration reactions [4], so higher coefficients K were observed, especially when no additional water was provided during the first seven days of the exposure period. In addition to this, it is important to emphasize that after 180 hardening days, the CEM III samples showed lower coefficient K values than CEM I ones for all the environment and curing conditions studied. This would indicate the beneficial effect of slag addition in relation to the reduction of the ingress of water in cement-based materials, even when they are exposed to a real in situ Mediterranean climate environment. As has been explained over the discussion of microstructure characterization results, this good performance could be related to the additional solid phases formation as products of slag hydration [12].

The effective porosity has the physical meaning of the volume fraction accessible for water and, as a consequence, for aggressive species, such as chlorides [39]. The effective porosity results (see Figure 8) were very similar to those noted for capillary suction coefficient K, so the previous discussion of that parameter would be also valid for the effective porosity. However, for both studied cements, practically no differences in effective porosity values were observed between the real environment exposure with and without curing, while this difference was more noticeable for coefficient K. Lastly, the effective porosities for CEM III samples were lower than those observed for CEM I ones, regardless of exposure environment. This would mean that the volume of pores accessible for water was smaller when slag cement was used, improving the durability of concretes hardened in the Mediterranean climate conditions in the short-term.

The study of the resistance against chloride ingress of concretes is important, because chlorides are one of the aggressive ions which can produce the corrosion of reinforcing elements. In this work, the chloride ingress resistance of slag and OPC concretes has been analyzed through the non-steady-state chloride migration coefficient D_NTB_. This coefficient was reduced with time for all the studied cement types and conditions (see Figure 9) and at early ages it showed higher values for the samples exposed to the real in situ environment without curing, which could be related to the lower water availability of that condition, as has been already explained. The lowest migration coefficients were noted for CEM III samples, independently of the environmental and curing conditions. This result would agree with many studies [14,17,18,19], which have demonstrated that the use of slag cement entails a substantial improvement in chloride ingress resistance. On one hand, the higher refinement of microstructure produced by the slag compared to pure clinker [12,15,19] could explain the improvement of resistance against chloride ingress observed for CEM III concretes. On the other hand, this good resistance of CEM III concretes against chloride ingress at earlier times and when they were exposed to non-optimum environments, which produced a lower microstructure refinement, could also be explained due to the higher binding capacity of slag cement, in comparison with ordinary Portland cement. This binding capacity is due to the high content of calcium aluminates brought by the slag [40].

Regarding the carbonation front depth results (see Figure 10), until 90 days the CEM I and III samples showed very similar values of this parameter for each condition. Nevertheless, at 180 days the slag concretes hardened in the real in situ Mediterranean climate environment (with and without 7 days of additional curing) presented higher carbonation front depths compared to CEM I concretes. This result would also be in keeping with several authors [12,17], which have noted that slag cement concretes would develop a higher carbonation rate due to their lower content in portlandite, compared to OPC ones. In spite of that, as has been observed in the already discussed results of this work, this lower carbonation resistance seems not to have a deleterious effect on the durability of studied slag concretes. Lastly, the additional seven-day curing provided to part of the samples exposed to the real environment had scarce influence in the carbonation front depths, at least throughout the studied time period.

In general, the results of durability-related properties analyzed in this work showed coincidences with those obtained for microstructure characterization, and they would suggest that concretes prepared using slag cements could have a good durability performance in the short-term when they are exposed to a real in situ Mediterranean climate environment. Moreover, their behavior could be similar or even better compared to concretes made with ordinary Portland cement.

### 4.3. Compressive Strength

At the majority of testing ages, for all studied conditions, the highest compressive strengths corresponded to CEM III samples, which could mean that slag cement concretes could have a good mechanical behavior in the short-term when they are kept under a real in situ Mediterranean climate environment (see Figure 11). Despite that, this non-optimum exposure environment entailed a reduction of compressive strength values for slag cement concretes in comparison with the optimum laboratory condition. This would agree with the microstructure and durability-related results of this research, and would show the higher sensitivity of slag hydration to lower environmental temperature and relative humidity, as already explained [4,21,22]. For CEM III samples exposed to the real environment and cured during the first seven hardening days, the compressive strength development was higher in the very short-term, due to the additional water supply, which would favor the slag hydration [4,21], although at 180 days the compressive strengths of slag concretes hardened in the real environment are similar, regardless of this complementary curing. This effect of the additional 7-day curing in the compressive strength was more noticeable for CEM I concretes, reaching this parameter similar values at 28 and 90 days for cured specimens exposed to real environment than for those kept in the optimum laboratory condition.

## 5. Conclusions

The main conclusions that can be drawn from the results previously discussed can be summarized as follows:

- The lower average environmental temperature and relative humidity of the real in situ Mediterranean climate condition seems to reduce the microstructure and service properties development rate for the studied concretes, probably due to the slowing down of the clinker and slag hydration caused by this non-optimal condition.
- The evolution of microstructure parameters observed for the slag cement concretes kept under the different environmental conditions studied, would reveal a higher sensitivity of slag hydration to hardening temperature.
- The additional seven-day curing, provided to a part of the samples exposed to the real in situ Mediterranean climate environment, overall improved the microstructure and service properties of the studied materials at shorter ages, although it hardly had an influence in their results at 180 days.
- In general, the pore network of slag cement concretes was more refined than that observed for CEM I ones, which could be related to the additional solid phases formed as products of slag hydration.
- The capillary suction coefficient and the effective porosity results would suggest that the slag addition could have a beneficial effect in relation to the reduction of the ingress rate of water in cement-based materials and the volume of pores accessible for water, even when they are exposed to a real in situ Mediterranean climate environment.
- The lowest non-steady state chloride migration coefficients were noted for CEM III samples, independently of the environmental and curing condition. This could be related to the pore network refinement produced by the slag compared to clinker and by the higher binding capacity of slag cement.
- At 180 days, the slag concretes hardened in the real in situ Mediterranean climate environment (with and without seven days of additional curing) showed higher carbonation front depths compared to CEM I concretes. Despite that, this has not produced a reduction on the durability and mechanical strength performance of the studied slag concretes.
- At the majority of testing ages and for all studied conditions, the highest compressive strengths corresponded to CEM III samples. This result would mean that slag cement concretes could have good mechanical behavior in the short-term when they are kept under a real in situ Mediterranean climate environment.
- Considering the results obtained in this research, concretes prepared using a sustainable cement with high content of slag and exposed to a real in situ Mediterranean climate environment, show good service properties in the short-term (180 days), which would be similar or even better compared to concretes made with ordinary Portland cement.

## Figures and Tables

**Table 1:** Components of the commercial cements used.

Component	CEM I		CEM III	
	UNE-EN 197-1 Standard [27]	Manufacturer Data ^1^	UNE-EN 197-1 Standard [27]	Manufacturer Data ^1^
Cement	95–100%	95%	20–34%	31%
Limestone	-	5%	-	-
Blast-furnace slag	-	-	66–80%	69%

^1^ Specific percentage of each component usually used according to the manufacturer.

**Table 2:** Dosage used for the preparation of concrete.

Component	Kg/m^3^
Cement	350
Coarse aggregate 4–6	489.5
Coarse aggregate 6–12	714
Sand	662.75
Water	175
Plasticiser	5.25
